# Mass drug administration should be implemented as a tool to accelerate elimination: against

**DOI:** 10.1186/s12936-019-2907-7

**Published:** 2019-08-22

**Authors:** Kamini Mendis

**Affiliations:** Colombo 5, Sri Lanka

**Keywords:** Malaria, Mass drug administration, Malaria elimination

## Abstract

In most malaria situations, mass drug administration (MDA) will result in a rapid reduction in the incidence and prevalence of malaria in the target population. However, due to practical reasons MDA hardly ever achieves coverage of the entire population and, therefore, will leave residual malaria infections in the population, from which malaria transmission can be resumed. Depending on the degree of access to prompt diagnosis and treatment and to effective vector control in the area, previous levels of incidence and prevalence will eventually be reached after MDA. It is, therefore, imperative that coverage with these interventions is ensured if MDA is to be implemented. Both effective vector control and access to treatment in combination will also reduce the malaria incidence and prevalence in an area, *albeit* more slowly than MDA. MDA’s role in elimination has to be considered in relation to the following: (1) MDA is logistically difficult, ethically questionable and may evoke parasite resistance to the medicines being used, (2) MDA will only accelerate elimination by reducing the starting number of infections, but that (3) it will be of no benefit to elimination unless both effective vector control and good access to treatment are in place. All malaria elimination efforts have, and will, succeed with good access to treatment, effective vector control, and case surveillance and response systems, and most have not, and will not require MDA. The role of MDA in elimination, if any, will be limited to some very specific situations—small foci of high transmission within a larger area which has made progress towards elimination, to which the former constitutes a continuing source of parasites and, therefore, could jeopardize the elimination effort in the larger area. Elimination of malaria needs not only to be achieved but also be sustained. This is particularly challenging in tropical countries where the risk of re-introduction is high. The haste to eliminate malaria using MDA must be balanced by investment of time and effort to establish effective vector control programmes, and case surveillance and response systems based on diagnosis and treatment services, which are core requisites for achieving elimination, and the latter for sustaining it.

## Background

Mass drug administration (MDA) is taken here to mean the administration of a full dose of anti-malarial treatment, irrespective of the knowledge of symptoms or presence of infection, to an entire population in a given area, except those in whom the medicine is contraindicated [1]. Thus, in addition to those that may harbour a malaria infection, the medicine will be administered to persons who do not have a malaria infection, who, in most situations are likely to constitute the majority.

## The effect of mass drug administration on elimination

When successfully conducted, MDA will, in infected persons to whom the medicine(s) was administered, eliminate a malaria blood infection, or hypnozoites in the case of relapsing *Plasmodium* species, or both, depending on whether it uses blood schizonticidal medicines, or a anti-hypnozoite medicine, such as primaquine, or both, respectively. As reported in most [2–4], but not all [5] situations this will rapidly reduce the incidence and prevalence of malaria in the area.

How this could help an elimination effort, however, needs more careful consideration. Central to this question is the fact that MDA programmes cannot achieve 100% coverage of the population in the given area, for a variety of reasons—some individuals not being accessible at the time of the campaign, refusal to take medicine by some, and those in whom the medicine is contraindicated. Thus, after even a very successful MDA programme, there will be residual parasite infections in the area. In the case of an elimination situation, importation of new infections by the passage of infected people into the area will also constitute a source of parasites.

**Fig. 1:**
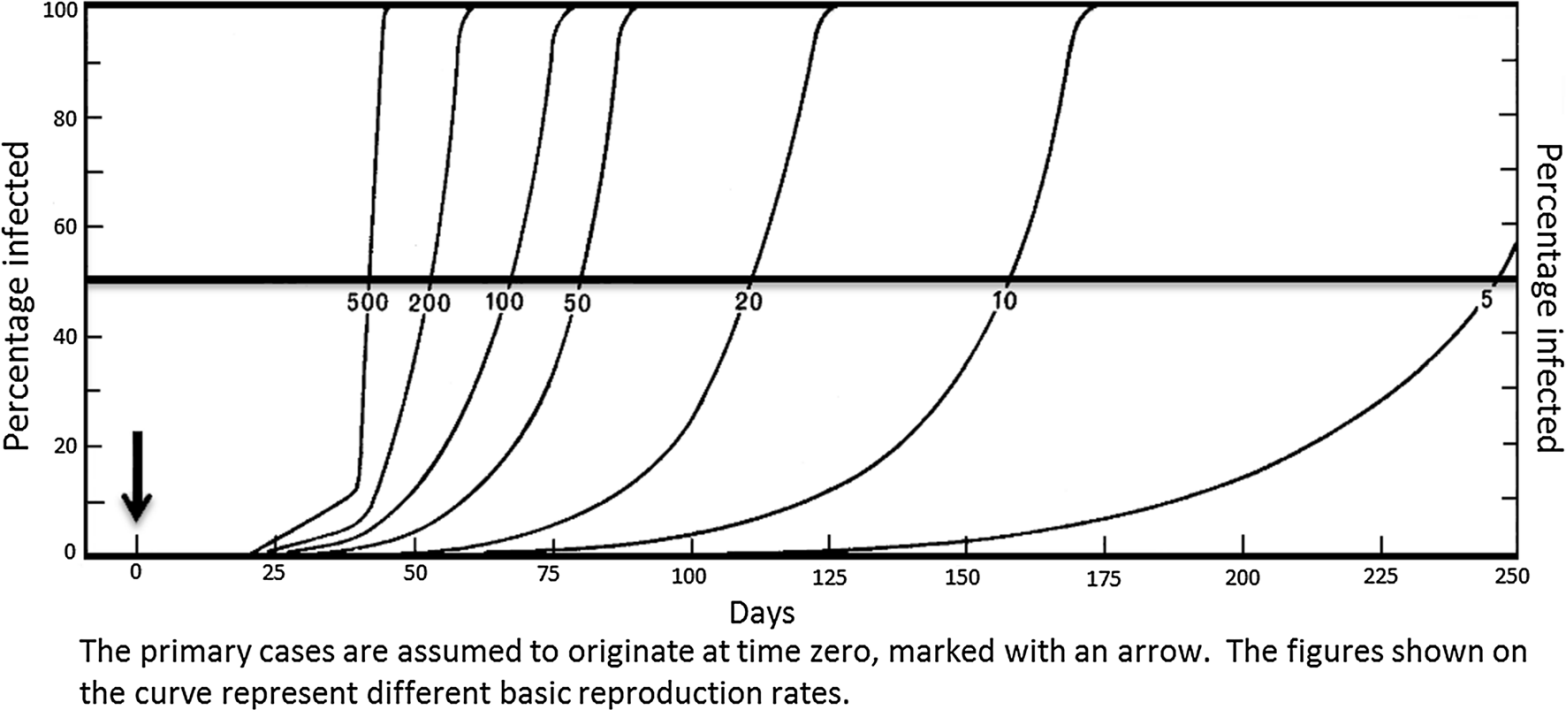
Probable growth in infection rates in *Plasmodium vivax* epidemics (incubation interval 20 days) from small origins (0.1% of the population), showing influence of basic reproduction rate (Reproduced from Macdonald [6])

**Fig. 2:**
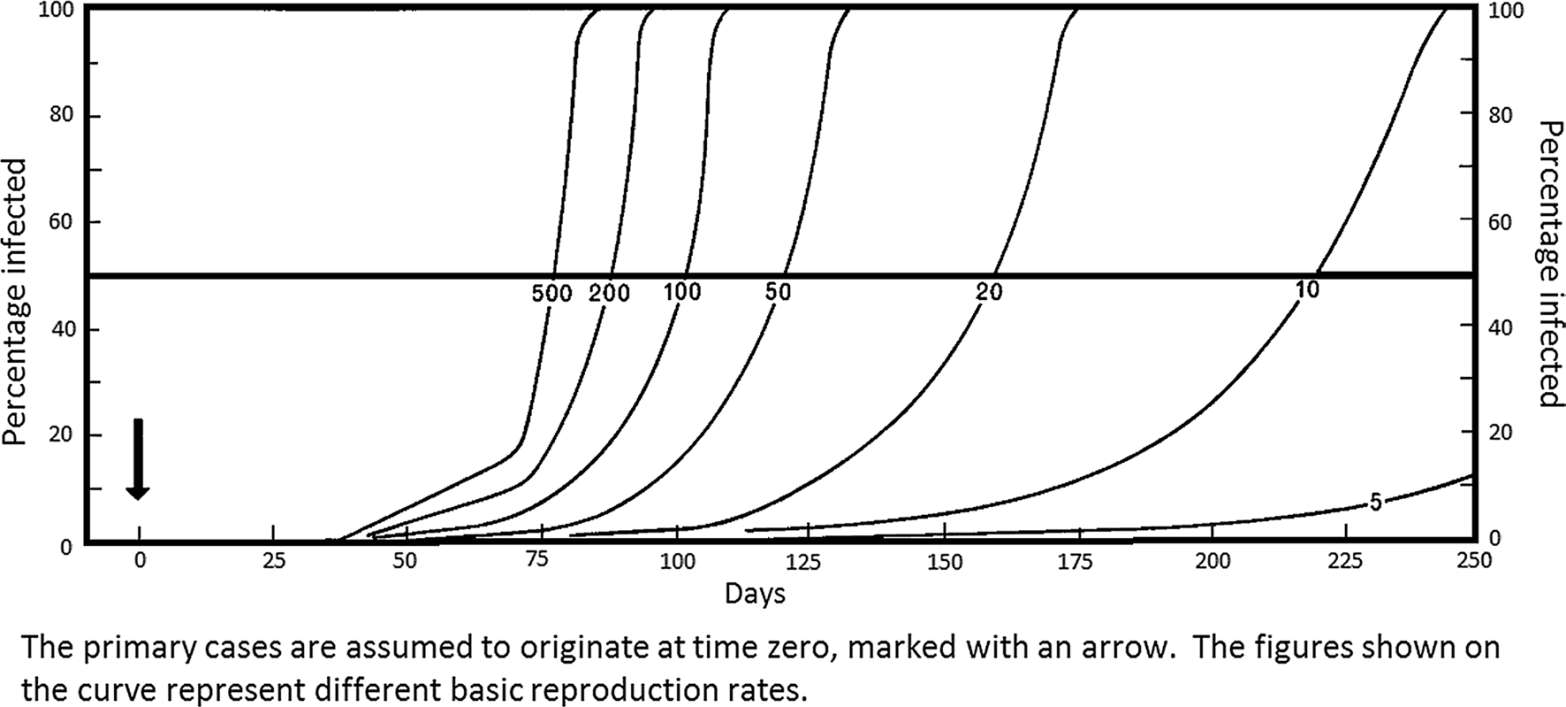
Probable growth in infection rates in *Plasmodium falciparum* epidemics (incubation interval 20 days) from small origins (0.1% of the population), showing influence of basic reproduction rate (Reproduced from Macdonald [6])

In malaria endemic situations where MDA has been applied, transmission can resume or an epidemic can arise starting from a very small number of infected people—those overlooked by the MDA programme or cases imported to the area, and the case numbers will increase depending on the prevailing basic reproduction rate of malaria as shown in Figs. 1, 2 for *Plasmodium vivax* and *Plasmodium falciparum*, respectively [6].

The basic reproduction rate of malaria which will determine how long it would take for transmission to return to previous levels, is governed entirely by the following factors:

1. The mean duration of infectivity in a primary case.
2. The density of mosquitoes in relation to man.
3. The frequency with which the vector bites man.
4. The longevity of the mosquito.

MDA will not affect any of these factors in the residual state of malaria. The basic reproduction rate will, however, be affected by the following interventions:

1. Access to diagnosis and effective treatment of malaria cases—this will affect the mean duration of infectivity in a primary case (factor (1) of above);
2. Effective vector control interventions—e.g., IRS, LLINs, larval control—these will affect factors (2), (3), and (4) above, i.e. the density of mosquitoes in relation to man, the frequency with which the vector bites man and the longevity of the mosquito. In quantitative terms effective vector control will have the greatest impact on the basic reproduction rate.

When there are a few residual cases of malaria in the area, as there will be after MDA, depending on the degree of access to diagnosis and treatment and vector control in the area, transmission will be resumed from a residual case(s). The rate at which the number of cases will increase and, therefore, the time taken to reach previous endemicity levels will depend on the degree of coverage with these two interventions.

MDA will greatly reduce the starting parasite reservoir, and to this extent accelerate elimination, provided, that good access to treatment and coverage with effective vector control can be provided simultaneously in the area. If not, MDA would have only a transient effect of reducing malaria prevalence and incidence, and it may, in fact, detract from the elimination programme by the sheer magnitude of effort and cost that a MDA programme requires. The published literature is rife with reports of successful MDA programmes in different endemicity situations, markedly reducing the malaria incidence and prevalence in the immediate aftermath. Although few if any have reported on the longer term follow-up [2], it is common knowledge that in all except a rare instance which was already in an elimination programme [7], malaria eventually returned to previous endemic levels.

## Accelerating malaria elimination *versus* sustaining zero transmission after elimination

It is important to note that good access to treatment and coverage with vector control in the area, will, in themselves, reduce the parasite prevalence, *albeit* more slowly than MDA. The author’s experience several decades ago with an isolated village in a Southeast Asian country where the prevalence of malaria was found to exceed 20% serves well to illustrate this point. It was an unusual situation for that country, caused by the remote village being overlooked by the health services. Within 6 weeks of establishing a makeshift clinic with a microscopist and anti-malarial medicines within the village and instituting standard vector control operations, the prevalence decreased to less than 1%.

This then shifts the central question to, ‘what specifically is the need to accelerate elimination, if this is all that MDA will do, provided of course that other interventions are well in place?’ Eliminating malaria from a country makes little sense unless it can be sustained. Sustaining zero transmission in countries that are now approaching elimination can be quite challenging, more so now than in the past, because most countries that are left with malaria and aim to eliminate the disease at present are in the tropical belt, where the risk of re-introduction is high. Under these conditions, fundamental to maintaining zero transmission is a robust and sustainable case surveillance and response system, based on diagnosis and treatment, which when combined with vector control will reduce malaria prevalence rates in an area. In situations where there is both good access to diagnosis and treatment and good coverage with vector control, which an eliminating country must have, one would not expect to find high parasite prevalence that requires intervention with MDA. The converse is also true—a high parasite prevalence in an area which would require intervention with MDA, is only a reflection of ‘poor’ malaria control, meaning the lack of an effective diagnosis and treatment service and vector control, a situation which is not yet ready to move to an elimination phase of operations. The haste to achieve elimination must be tempered by need to invest time and effort to build systems to also sustain elimination.

There is nevertheless, possibly one isolated situation in which MDA could benefit an ongoing elimination programme. This would be in a confined geographical area which is a focus of ongoing transmission at sub-district level, within a larger region, such as a country or district which is nearing elimination, the former having a high prevalence of malaria with chronic asymptomatic blood infections which could infect mosquitoes. An example would be a multi-drug resistant focus in the Greater Mekong Subregion, where countries are achieving major reductions in malaria incidence through elimination programmes. MDA in this situation may be justified if the area in question acts as a parasite reservoir for adjacent areas which are nearing elimination and will, therefore, by its neighbouring location, jeopardize the achievement of elimination in the larger area—a risk that could be mitigated by rapidly reducing the malaria incidence and prevalence in the area in question using MDA. Since the adjacent areas are nearing elimination, the rapid reduction of incidence and prevalence by MDA in the focus could also be a boost for moving this area into elimination, but here again, only if access to effective treatment and vector control are well established in the focus. Presumably neither were, previously, which led to the high prevalence.

A 2015 report of the World Health Organization [1] on the wider role of MDA in malaria includes a specific and guarded recommendation that “*MDA can be considered in the elimination of P. falciparum malaria in areas approaching interruption of transmission where there is good access to treatment, effective implementation of vector control and surveillance, and a minimal risk of re*-*introduction of infection*”. With the exception of the situation described above, there can be very few instances where this recommendation would apply. This is because areas approaching elimination, where robust diagnosis and treatment systems as well as vector control will be in operation, are not likely to have high prevalence rates that call for intervention with MDA.

It is a widely held misconception that for elimination to be achieved, the last parasites in the area, which are likely to reside in asymptomatic cases, must be actively pursued and eliminated. This has given further credence to the view that MDA has an important role in accelerating elimination. In elimination scenarios, asymptomatic infections are nearly always a small proportion of all infections, most of which would be symptomatic. With decreasing transmission rates during the elimination drive such asymptomatic infections, if any, eventually ‘die out’. It has been the experience of almost every country that eliminated malaria to date that elimination was achieved without active intervention targeting asymptomatic cases.

## Conclusion

MDA will achieve what good access to treatment and effective vector control will achieve, only faster. The latter two interventions are core requirements to achieve and sustain elimination—they are essential. Besides, without them, the effect of MDA will be all but fleeting. The wisdom of investing in an intervention, such as MDA, which is logistically arduous, ethically questionable and associated with a risk of inducing drug resistance, only to gain a little time to reach the goal of elimination, is highly questionable. This is especially so, given that sustaining the achievement will requires many decades, if not centuries of effort to maintain a case surveillance and response system, possibly even until global eradication is reached.

## Data Availability

Not applicable.

## Abbreviations

MDA: mass drug administration
IRS: indoor residual spraying
LLIN: long-lasting insecticidal nets
WHO: World Health Organization
